# Localization of sesquiterpene formation and emission in maize leaves after herbivore damage

**DOI:** 10.1186/1471-2229-13-15

**Published:** 2013-01-30

**Authors:** Tobias G Köllner, Claudia Lenk, Christiane Schnee, Sabrina Köpke, Peter Lindemann, Jonathan Gershenzon, Jörg Degenhardt

**Affiliations:** 1 Max Planck Institute for Chemical Ecology, Hans-Knöll-Strasse 8, D-07745, Jena, Germany; 2 Institute of Pharmacy, Martin-Luther-University Halle-Wittenberg, Hoher Weg 8, D-06120, Halle/Saale, Germany; 3 Current address: Friedrich Löffler Institute, Naumburger Strasse 96a, Jena, 07743, Germany

**Keywords:** Zea mays L., Poaceae, Maize, Sesquiterpenes, Volatiles, Terpene biosynthesis, Terpene synthase, Herbivore-induced terpene formation, Jasmonic acid, Solid-phase microextraction (SPME)

## Abstract

**Background:**

Maize (*Zea mays* L.) leaves damaged by lepidopteran herbivores emit a complex volatile blend that can attract natural enemies of the herbivores and may also have roles in direct defense and inter- or intra-plant signaling. The volatile blend is dominated by sesquiterpenes of which the majority is produced by two herbivore-induced terpene synthases, TPS10 and TPS23. However, little is known about the pattern of volatile emission within maize leaves.

**Results:**

In this study, we restricted herbivore feeding to small sections of the maize leaf with the aim of determining the patterns of volatile sesquiterpene emission throughout the damaged leaf and in neighboring leaves. Sesquiterpene volatiles were released at high rates from damaged leaves, but at much lower rates from neighboring leaves. Release was restricted to the site of damage or to leaf sections located apical to the damage, but was not seen in sections basal to the damage or on the other side of the midrib. The emission pattern correlated well with the transcript pattern of the respective sesquiterpene synthase genes, *tps10* and *tps23*, implying that biosynthesis likely occurs at the site of emission. The concentrations of jasmonic acid and its leucine derivative were also elevated in terpene-emitting tissues suggesting a role for jasmonates in propagating the damage signal.

**Conclusions:**

In contrast to other defense reactions which often occur systemically throughout the whole plant, herbivore-induced sesquiterpene production in maize is restricted to the wounding site and distal leaf parts. Since the signal mediating this reaction is directed to the leaf tip and cannot propagate parallel to the leaf axis, it is likely connected to the xylem. The increasing gradient of volatiles from the tip of the leaf towards the damage site might aid herbivore enemies in host or prey finding.

## Background

The emission of volatiles by plants allows them to interact with other organisms at a distance. For example, many plant species emit floral volatiles which have diverse functions in pollinator attraction and repulsion [1]. Volatiles are also released from vegetative plant organs, especially after herbivore damage. Vegetative volatiles can function as attractants for enemies of herbivores [2] and have been suggested to serve as direct defenses against herbivores [2], as defenses against pathogens [3], as protectants against abiotic stress, and as signals in intra- and inter-plant communication [4]. However, some of these roles are only poorly studied. More knowledge about precisely where volatiles are emitted within a leaf might reveal more about their roles.

During the last two decades maize (*Zea mays* L.) has been established as a model system for studying herbivore-induced vegetative volatiles. Maize leaves damaged by lepidopteran larvae emit a complex blend of volatiles that can be utilized by parasitoids of the herbivore to locate their potential hosts on the infested plant [5]. The parasitoids oviposit on or into the host which continues to feed, but often at reduced levels, and eventually dies without reaching the adult stage [6,7]. Recruitment of herbivore enemies can thus be beneficial for the plant and has been termed “indirect plant defense”. The volatile blend of herbivore-damaged maize leaves consists of three classes of compounds: 1) mono-, sesqui- and homoterpenes produced by the isoprenoid pathways, 2) green-leaf odors such as saturated and unsaturated six-carbon alcohols and esters derived from the lipoxygenase pathway and 3) aromatic compounds like indole, methyl anthranilate and methyl salicylate generated by the shikimate/tryptophan pathway [5,8]. The quantitative and qualitative composition of this blend varies between maize cultivars but is usually dominated by sesquiterpenes [8-11]. While the majority of the sesquiterpene volatiles is released from the herbivore-damaged leaf itself, low levels of volatile emission have also been observed from adjacent leaves [12]. The composition of the blend from adjacent leaves is different from damaged leaves in that it lacks most sesquiterpenes but still contains the monoterpene linalool and the homoterpenes (*3E*)-4,8-dimethyl-1,3,7-nonatriene (DMNT) and (*3E7E*)-4,8,12-trimethyl-1,3,7,11-tridecatetraene (TMTT) [12].

Previous studies with maize have shown a strong correlation between monoterpene and sesquiterpene emission and biosynthesis [10], suggesting that compounds are released soon after synthesis without any long-term storage. But the relationship between emission and biosynthesis has never been studied within individual leaves. The majority of herbivore-induced sesquiterpenes of maize is produced by two terpene synthases (TPS) which both convert the farnesyl diphosphate (FPP) substrate into multiple, structurally diverse products. The terpene synthase TPS10 forms (*E*)-*α*-bergamotene and (*E*)-*β*-farnesene along with thirteen minor sesquiterpenes in herbivore-damaged leaves [13]. The efficacy of TPS10 sesquiterpenes for indirect defense against lepidopteran larvae has been tested with Arabidopsis plants overexpressing TPS10. After an initial learning experience, the TPS10 volatiles were utilized by the parasitic wasp *Cotesia marginiventris* to find their lepidopteran host [13]. The herbivore-induced sesquiterpene synthase TPS23 forms mostly (*E*)-β-caryophyllene which can also attract parasitic wasps to leaf-feeding herbivores [14]. In addition, TPS23 is induced in roots after damage by the western corn rootworm (*Diabrotica virgifera virgifera*) where the (*E*)-β-caryophyllene product attracts entomopathogenic nematodes to the attacking herbivore [14,15]. While nearly all maize cultivars produce the TPS10 products (*E*)-*α*-bergamotene and (*E*)-*β*-farnesene, the TPS23 product (*E*)-β-caryophyllene is only formed in European maize lines [8,14]. Apparently, many maize lines derived from North American breeding programs have lost the capability to express TPS23 [14]. Beside TPS10 and TPS23, a further terpene synthase was recently described to be involved in vegetative volatile biosynthesis in maize. This enzyme, TPS8, is constitutively expressed in nearly all plant organs and seems to be involved in pathogen defense (Fontana A, Köllner TG, Schnee S, Reichelt M, Rosenberger D, Luck K, Gershenzon J, Degenhardt J: A complex blend of volatile sesquiterpenes produced by maize terpene synthase 8 (TPS8) is active against a necrotrophic fungus *in planta*. submitted).

Despite all of the available information about the chemical and molecular bases of terpene emission in maize, we know little about its location within individual leaves. More knowledge of where volatiles are made and emitted in maize leaves would not only shed more light on their function, but also increase our understanding of what controls synthesis and emission. In this study we have investigated the pattern of sesquiterpene emission and biosynthetic gene expression in herbivore-damaged vs. control leaves and at the site of herbivory vs. sections of the rest of the leaf. To detect the small amounts of sesquiterpenes emitted by individual leaf parts, we analyzed the volatiles released from macerated tissues and used them as an estimate for natural sesquiterpene emission. Both gene expression and emission data indicate a directed movement of a damage-induced signal toward the apex of the leaf and not across the midrib. Analysis of defense hormone levels within the leaf suggests the involvement of jasmonates in the signaling process.

## Results

### Sesquiterpene production is induced in herbivore-damaged leaves, but only at low levels in neighboring control leaves

The response of the leaves of maize cultivar B73 to herbivore attack was investigated by measurement of both sesquiterpene release and the transcript level of TPS10, the most important enzyme in volatile sesquiterpene biosynthesis in herbivore-damaged leaves. Since there is no standard method sensitive enough to measure head-space volatiles from small areas of plant tissue, we macerated leaf samples in liquid nitrogen and analyzed the release of terpenes from the resulting powder using solid-phase microextraction (SPME). A preliminary experiment carried out on entire plants comparing the maceration method with conventional head-space volatile collection revealed that the amount of sesquiterpenes released from total leaf powder and collected using a SPME fiber was directly correlated with the amount of head-space sesquiterpenes emitted from intact plants (Additional file 1: Figure S1) demonstrating the value of the maceration method as a good proxy for natural sesquiterpene emission.

**Figure 1:**
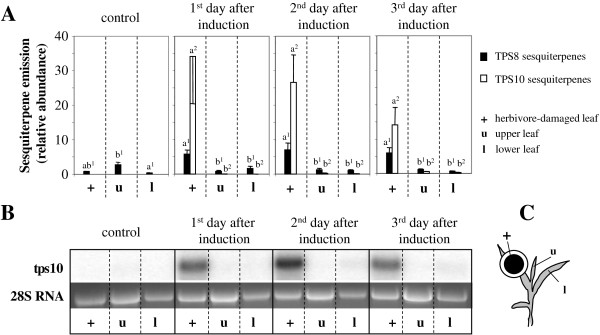
**Variation in volatile sesquiterpene emission and terpene synthase transcript levels between herbivore-damaged vs. neighboring control leaves.** (**A**) Terpene volatiles emitted from herbivore-damaged leaves (+), the leaves immediately above the damaged ones (u) and the leaves immediately below damaged ones (l) of the maize inbred line B73 collected one, two or three days after induction with *Spodoptera littoralis* caterpillars. Volatiles were collected using SPME and analyzed by GC-MS. Sesquiterpenes were classified in two groups: TPS10 sesquiterpenes including (*E)*-*α-*bergamotene, (*E*)-*β*-farnesene, and *β*-sesquiphellandrene and TPS8 sesquiterpenes including *α*-copaene, (*E*)-*β*-caryophyllene, germacrene D, and δ-cadinene. Means and standard errors (n=3) of three independent measurements are given. Different letters with the same numbered superscripts (e.g. a^1^ and b^1^) indicate statistically significant (p<0.05) differences between the herbivore-damaged leaf, the upper leaf and the lower leaf for each terpene group and time point. (**B)** Expression of *tps10* transcript in *S. littoralis*-damaged and neighboring control leaves over the same time course. RNA was prepared from leaf samples as described in the methods, blotted and probed with a radio-labeled *tps10-*specific PCR-fragment. Images were obtained with a phosphor imager. (**C)** Experimental setup showing cage for *S. littoralis* larvae on herbivore-damaged leaf (+) in relation to neighboring control upper leaf (u) and lower leaf (l).

The volatile sesquiterpenes emitted from the leaves can be categorized in two groups according to their biosynthetic origin (Figure 1). The first group are products of the herbivore-induced TPS10, including the sesquiterpenes (*E*)-*α-*bergamotene, (*E*)-*β*-farnesene and *β*-sesquiphellandrene [13], while the second group are the products of TPS8, a multiproduct sesquiterpene synthase constitutively expressed in all organs of the maize variety B73, including *α*-copaene, (*E*)-*β*-caryophyllene, germacrene D and δ-cadinene (Fontana A, Köllner TG, Schnee S, Reichelt M, Rosenberger D, Luck K, Gershenzon J, Degenhardt J: A complex blend of volatile sesquiterpenes produced by maize terpene synthase 8 (TPS8) is active against a necrotrophic fungus *in planta*. submitted). The (*E*)-*β*-caryophyllene synthase TPS23 is not involved in (*E*)-*β*-caryophyllene formation in B73 since *tps23* is not expressed in this variety [14].

Herbivore-damaged leaves emitted TPS10 and TPS8 terpenes at much higher rates than control leaves. The control leaves released only relatively low amounts of TPS8 compounds (Figure 1). Neighboring, undamaged leaves of herbivore-induced plants emitted only trace amounts of TPS10 products along with levels of TPS8 terpenes that were comparable to those of control plants. Transcript levels of *tps10* were determined by RNA hybridization. The gene was highly expressed in damaged leaves, but no transcripts could be detected in neighboring control leaves. This pattern is similar to that of the TPS10 volatile measurements and indicates no systemic induction of volatiles beyond the herbivore-damaged leaf in maize line B73.

### Terpene emission in maize leaves is restricted to the area between the site of damage and the leaf apex

**Figure 2:**
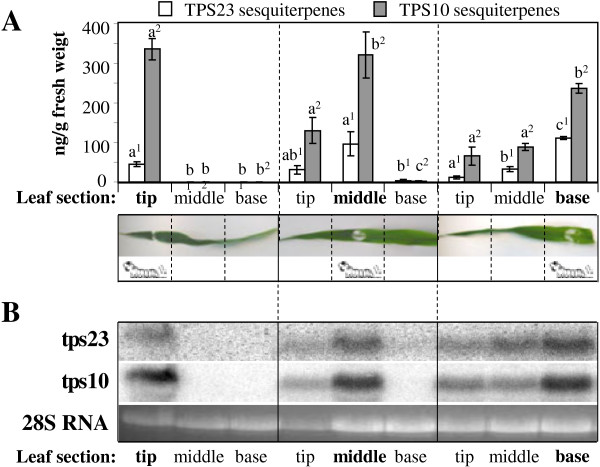
**Variation in volatile sesquiterpene emission and terpene synthase transcript levels among different sections of a herbivore-damaged leaf. (A)** Emission of (*E*)-*α*-bergamotene and (*E*)-*β*-farnesene (TPS10 products) and (*E*)-*β*-caryophyllene (TPS23 product) from different leaf sections of maize variety Delprim after herbivory restricted to one section (in bold letters). One *S. littoralis* larva was enclosed for seven hours on the tip, the middle or the base, respectively, of the second fully expanded leaf. For terpene analysis, the leaves were divided into three equal parts (tip, middle, base) and volatiles were collected from tissue powder using SPME and analyzed by GC-MS. Data are means of four replications ± SE. Different letters with the same numbered superscripts (e.g. a^1^ and b^1^) indicate statistically significant (p<0.05) differences between the volatile measurement of the single leaf sections for each treatment and each sesquiterpene type. **(B)** Transcript levels of *tps10* and *tps23* in different leaf sections after herbivory restricted to one section. RNA was prepared from an aliquot of the tissue powder used for volatile analysis (see above). The samples were blotted and probes specific for *tps10* and *tps23* were used for hybridization. The 28S RNA is shown as a loading control on an ethidium bromide stained agarose gel.

To analyze the spatial distribution of terpene emission around the site of herbivory, we measured the volatiles emitted from different leaf sections. These experiments used the maize variety Delprim which releases volatiles at higher rates than line B73 [8]. The base, middle, and apex of the second fully expanded leaf were exposed to *S. littoralis* larvae for seven hours. A small cage was used to restrict the damage to the respective leaf section. In contrast to the volatile blend emitted by B73 (Figure 1), the hybrid line Delprim produced (*E*)-*β*-caryophyllene from TPS23 but no TPS8 terpenes. After herbivore feeding on the leaf base, (*E*)-*α*-bergamotene and (*E*)-*β*-farnesene (TPS10) and (*E*)-*β*-caryophyllene (TPS23), were produced by all sections of the leaf (Figure 2A). However, herbivory restricted to the middle of the leaf led to the emission of sesquiterpenes from only the middle and upper sections of the leaf; no sesquiterpenes were released from the leaf base. After herbivore feeding on the leaf apex, sesquiterpenes were emitted exclusively from the upper section.

### Sesquiterpene synthases are expressed at the site of sesquiterpene emission

To analyze whether the sesquiterpenes found in the leaf distal to the damage site are produced *de novo* or transported from the site of wounding into these leaf tissues, we measured the transcript accumulation of the genes *tps10* and *tps23*, which encode the enzymes producing the herbivore-induced sesquiterpenes, in the different leaf parts. RNA hybridization demonstrated that the patterns for transcript accumulation of *tps10* and *tps23* (Figure 2B) were identical with those of their respective volatile products (Figure 2A). This suggests that (*E*)-*β*-caryophyllene, (*E*)-*α*-bergamotene, and (*E*)-*β*-farnesene are likely formed *de novo* at the site of emission by the action of the terpene synthases *tps10* and *tps23.*

### The signal mediating herbivore-induced terpene formation is not transported laterally across the midrib

**Figure 3:**
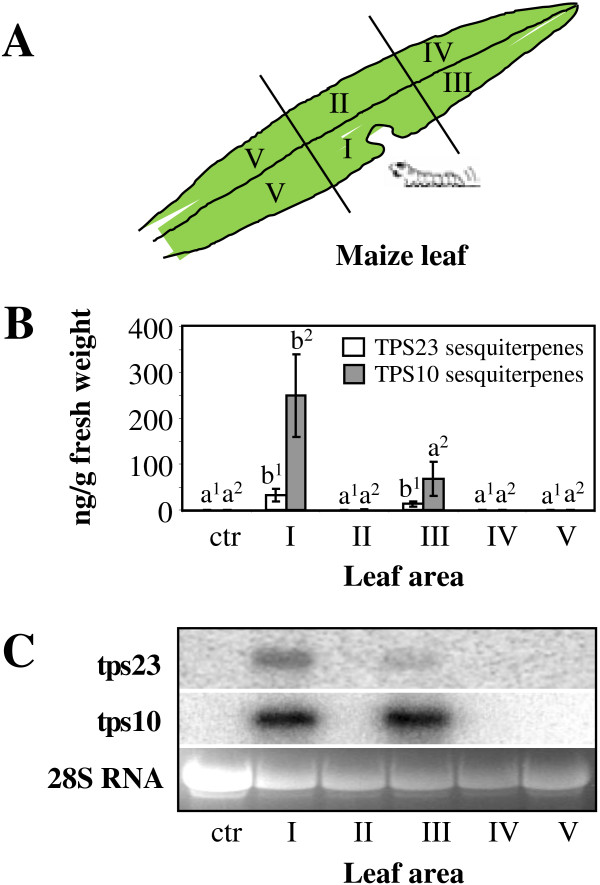
**Variation in volatile sesquiterpene emission and terpene synthase transcript levels between different sides of a leaf damaged by herbivory on one side. (A)** The cartoon illustrates the different leaf parts collected for volatile and transcript analysis. One *S. littoralis* larva was enclosed for seven hours on leaf part I of the second fully expanded leaf of maize variety Delprim. **(B)** Emission of (*E*)-*α*-bergamotene and (*E*)-*β*-farnesene (TPS10 products) and (*E*)-*β*-caryophyllene (TPS23 product) from different leaf parts after herbivory restricted to one section on one side. Volatiles were collected from tissue powder using SPME and analyzed by GC-MS. Data are means of four replications ± SE. Different letters with the same numbered subscripts (e.g. a^1^ and b^1^) indicate statistically significant (p<0.05) differences between the volatile measurement among the five distinct sections of one leaf for each set of sesquiterpene products. Control tissue (Ctr) was harvested from an undamaged leaf. **(C)** Transcript accumulation of *tps10* and *tps23* in different leaf parts after herbivory restricted to one section on one side. RNA was prepared from an aliquot of the tissue powder used for volatile analysis (see above). The samples were blotted and probes specific for *tps10* and *tps23* were used for hybridization. The 28S RNA is shown as a loading control on an ethidium bromide stained argarose gel.

Since herbivore-induced terpene formation is restricted to the site of damage and distal areas of the leaf, a signal must be transmitted from the damaged tissue to the leaf tip. To test whether this signal can propagate across the midrib perpendicular to the leaf axis, we measured the herbivore-induced sesquiterpenes (*E*)-*β*-caryophyllene, (*E*)-*α*-bergamotene, and (*E*)-*β*-farnesene as well as the transcript accumulation of the corresponding biosynthetic genes *tps10* and *tps23* separately on both sites of the midrib after herbivory restricted to only one side (Figure 3A). As observed previously, the tissues at the site of damage and distal to it emitted induced volatiles and accumulated transcripts of *tps10* and *tps23* (Figure 3B and C). However, only traces of volatiles and no transcripts could be detected in the corresponding tissues on the other side of the midrib (Figure 3B and C), suggesting that the signal is not transmitted laterally across the midrib.

### The accumulation pattern of jasmonic acid and its isoleucine conjugate correlates with the sites of terpene release

**Figure 4:**
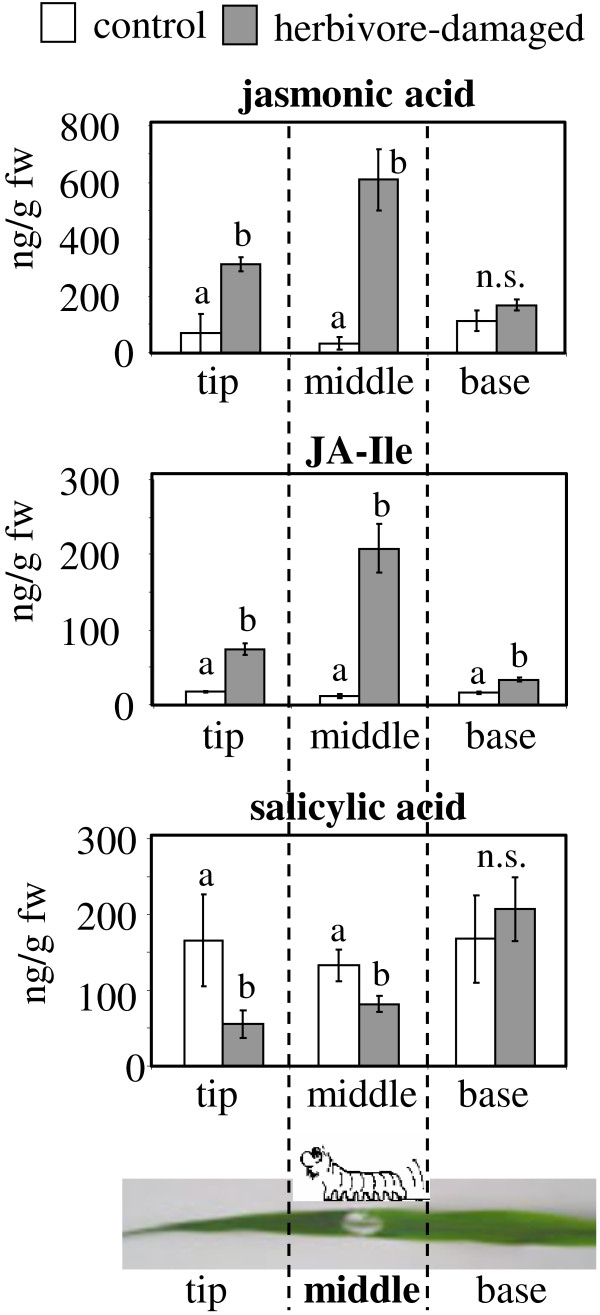
**Variation in defense hormone distribution among different sections of an herbivore-damaged leaf.** One *S. littoralis* larva was enclosed for seven hours on the middle of the second fully expanded leaf of maize variety Delprim. For plant hormone analysis, the leaves were divided into three equal parts (tip, middle, base) and hormones were extracted from tissue powder and analyzed by LC-MS. Data are means of four replications ± SE. Different letters indicate statistical significance between damaged and control plants for the tip, middle, and the base of one leaf.

The plant hormone jasmonic acid (JA) and its isoleucine conjugate (JA-Ile) are involved in the signaling of direct and indirect plant defenses against herbivores [16]. In maize it has been shown that the emission of volatile sesquiterpenes closely corresponds to the level of endogenous JA in the damaged leaf [17-19] and emission is inducible by the application of exogenous JA to excised as well as intact leaves [18,20]. To test whether jasmonates might play a role as mobile signals within the leaf, we measured the levels of JA and JA-Ile in the different parts of maize leaves after herbivore damage to the middle section of the leaf. The levels of JA and JA-Ile were strongly increased in herbivore-damaged leaves around the damage site and distally in the leaf tip relative to control leaves (Figure 4). Since the patterns of JA and JA-Ile concentration were similar to those of the terpene synthase transcript levels, terpene production might well be mediated by a JA-dependent signaling process. Another plant hormone involved in plant defense, salicylic acid (SA), is frequently involved in anti-pathogen responses and can act as a JA antagonist [21]. Herbivory resulted in slightly decreased levels of SA at the damage site and above in comparison to control leaves (Figure 4). There was no difference between SA levels in the bases of herbivore-damaged and control leaves.

### Herbivore-induced terpene biosynthesis takes place in the inner leaf tissues and not in the epidermis

**Figure 5:**
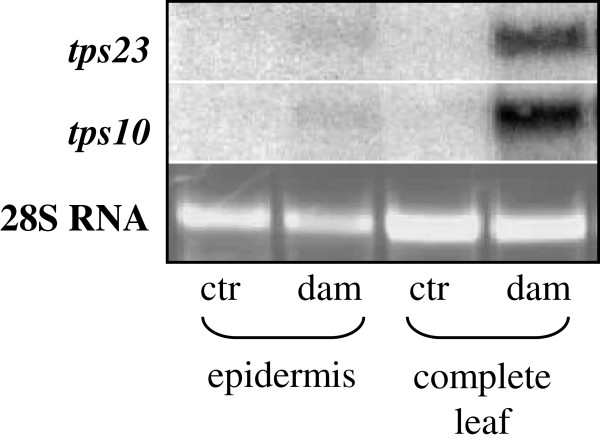
**Variation in transcript levels of *tps10 *and *tps23 *between epidermis and inner leaf tissues.** Samples from the isolated epidermis and complete leaf of maize variety Delprim were compared after herbivore damage (dam) and in undamaged control plants (ctr). The epidermis was carefully stripped from the leaves and RNA was prepared from epidermis tissue as well as from the complete leaf containing the epidermis and inner tissues. The samples were blotted and probes specific for *tps10* and *tps23* were used for hybridization. The 28S RNA is shown as a loading control on an ethidium bromide stained agarose gel.

Tissue and cell specific localization of volatile terpene biosynthesis could help to understand the regulation and ecological role of this biochemical pathway. Because localization experiments using antibodies specific for the recombinant terpene synthases TPS10 and TPS23 were unsuccessful (data not shown), we measured transcript accumulation of *tps10* and *tps23* in isolated epidermal tissue in comparison to the complete leaf to get insight into whether terpene biosynthetic rate differed among various leaf tissues. The epidermis was carefully stripped from the leaf surface using a sharp pair of tweezers. RNA was isolated from epidermal tissue powder and from leaf powder obtained from all tissues together. Hybridization experiments using specific probes for *tps10* and *tps23* showed that both gene transcripts accumulated in the whole leaf after herbivore damage, but could barely be detected in the epidermis. The weak bands in the damaged epidermis sample may just be caused by contamination of underlying tissue from the stripping procedure. These results suggest that transcript accumulation of *tps10* and *tps23* is localized in inner leaf tissues like mesophyll or bundle sheath cells and not in the epidermis (Figure 5).

## Discussion

Herbivore damage to specific plant organs frequently results in systemic defense responses, such as emission of volatiles from other parts of the plant [4] or the production of defensive proteins like protease inhibitors in adjacent organs [16]. However, here we report that the induction of sesquiterpene volatiles from the maize inbred line B73 damaged by *S. littoralis* is almost completely restricted to the damaged leaf and occurs at only trace levels in adjacent leaves. Within the herbivore-damaged leaf, emission was highest at the site of herbivore damage, declining slowly towards the upper part of the leaf, but was barely detectable towards the base of the leaf (Figure 2A). These results plus measurements of biosynthetic gene expression and jasmonate signaling compounds within the leaf have interesting implications for the physiology, function and regulation of terpene emission in maize.

The limitation of volatile sesquiterpene emission to the actual leaves damaged by herbivores and not to undamaged adjacent leaves fits with previous studies [12]. In general, when volatile emission after herbivory has been measured in a leaf-specific manner, many other plant species also show only localized release of sesquiterpenes and/or monoterpenes that is largely confined to the damaged leaves. For example, the emission of herbivore-induced sesquiterpenes from holm oaks is mostly restricted to herbivore-infested leaves [22]. The absence of a systemic response was also observed after wounding of transgenic Arabidopsis that contained an herbivore-inducible monoterpene synthase promoter-GUS fusion [23]. In contrast, the study of maize roots attacked by the larvae of *Diabrotica virgifera virgifera* did show systemic induction [24] with (*E*)-ß-caryophyllene emission being detected at the site of attack and in adjacent roots.

The emission of sesquiterpene volatiles from the site of damage and towards the leaf tip, but not towards the leaf base, will create a volatile gradient from the tip of the leaf increasing towards the damage site. For herbivore enemies that start their searching at the tips of the long leaves, this might aid in host or prey finding. If herbivore-induced sesquiterpenes function in other ways as signals to other plants or other parts of the same plant [4], release from the tip may also be valuable to increase the range of signal transmission. On the other hand, if volatiles act in direct defense against herbivores [2], their release from the damage site and apical, but not basal portions of leaves is not readily understandable unless feeding herbivores tend to move apically as they feed. One might expect increased emission from the leaf base since the presence of meristematic tissue likely makes it more valuable than the tip. However, greater emission from the tip may be an unavoidable result of a damage signaling pathway that is constrained to be directed toward the leaf apex rather than the base.

The localization of sesquiterpene formation to the inner leaf tissues of maize may reflect a link to photosynthesis. Constitutive and herbivore-induced terpene emission in plants typically follows a diurnal rhythm with an emission maximum during the day and emission minimum during the night [17,23,25-27]. Moreover, a previous study showed that volatile release from leaves of herbivore-damaged maize seedlings is dependent on light. In experiments with an artificial light–dark cycle comprising three repetitions of three hours light and three hours dark, Gouinguene and Turlings [28] demonstrated that the plants only released herbivore-induced volatiles during the light phases. In the present study, the transcripts of *tps10* and *tps23* were not found in the epidermis, but only in underlying leaf tissues. Given the fact that much of this underlying tissue is photosynthetic, the dependence of volatile terpene biosynthesis on light suggests that volatile formation in leaves occurs in photosynthetic cells. A finer, cell-specific localization of *tps* transcripts and measurements of terpene biosynthetic rate after application of specific photosynthetic inhibitors could help to understand more about the link between light and terpene emission. Sesquiterpene formation occurring in maize roots [24] does not appear to have a direct link to photosynthesis.

The close correlation of the site of terpene synthase expression in maize and the site of volatile terpene emission suggests that terpene products are formed *de novo* in the emitting tissues and are not transported from the damage site to other parts of the leaf, such as the tip. Previous experiments with radioisotopes established definitively that in cotton herbivore-induced, systemic volatiles are synthesized at the site of release [29]. To induce terpene synthase activity in distant parts of the leaf, a mobile chemical messenger must exist that moves from the site of damage to other parts of the plant. The first such systemic signaling compound, the oligopeptide systemin, was described in species of the Solanaceae [30]. However, the pattern of systemic damage signaling in maize is fundamentally different than that in the Solanaceae [31] and other dicotyledonous plants as the signal is only directed from the wounding site in an apical direction, but cannot cross the midrib. This signal path is much like that of xylem movement which conducts water and nutrients from the roots to above-ground organs, suggesting that the mobile damage signal moves through the xylem. It is notable that several plant hormones like abscisic acid, cytokinins and strigolactones are transported by the flow of the xylem [32].

The plant hormone JA and related jasmonates are perhaps the best known components of signal transduction pathways which trigger defense responses after herbivory, including the emission of volatiles in many plant species [16,33-35]. These compounds are known to act at a distance from the site of herbivore damage. Radio-labeled JA applied to leaves of *Nicotiana sylvestris* was transported to the roots where it is thought to increase nicotine biosynthesis in these organs [36]. Similarly, methyl jasmonate can function as a mobile signal in *N. tabacum* where it is transported via the phloem and the xylem to undamaged systemic leaves [37]. In the wound response of tomato, Wasternack and coworkers [38] concluded that JA, rather than the peptide systemin, acts as the mobile signal. The plant hormone JA could also be involved in mobile defense signaling in maize leaves, given that its accumulation is highest at the sites of highest volatile emission (Figure 4). A similar JA accumulation pattern has recently been reported by Engelberth and coworkers [39,40]. In both studies, herbivory on the middle part of a maize leaf was mimicked by mechanical wounding and subsequent application of either crude caterpillar regurgitant [39] or the elicitor N-linolenoyl-glutamine [40]. Both treatments resulted in high JA accumulation around the wounding site and in the leaf tip, but only trace accumulation in the leaf base. Furthermore, a transcript analysis of allene oxide synthase (AOS), an enzyme considered to catalyze the key step in JA biosynthesis [34], revealed that *AOS* expression was highest in the treated part of the leaf and the distal tissues [40]. Since the expression of AOS and other JA biosynthesis genes is known to be inducible by jasmonates, one can speculate that these compounds could also function as long-distance signals in maize, being transported via the xylem from the wounding site to apical leaf tissues where they induce their own biosynthesis as well as the expression of terpene synthase genes. However, the presence of other signaling compounds that activate JA biosynthesis locally after transport cannot be excluded at this time, and physical processes like wound-induced changes in hydraulic pressure [41] or an electrical signal [42] could also be involved. Further studies with labeled jasmonates and the chemical analysis of maize xylem sap might help to understand the complete nature of the signals that mediates terpene biosynthesis in this species.

## Conclusions

Our study showed that herbivore-induced sesquiterpene formation in maize is restricted to the wounding site and distal leaf parts. The resulting volatile gradient between the leaf tip and the site of damage might aid herbivore enemies in host or prey finding. Since the signal mediating this reaction is directed to the leaf tip and cannot across the midrib, it is likely connected to the xylem.

## Methods

### Plant and insect material

Seeds of the maize (*Zea mays* L.) hybrid line Delprim were kindly provided by Delley Samen und Pflanzen (Delley, Switzerland), and seeds of the inbred line B73 were obtained from KWS seeds (Einbeck, Germany). Plants were grown in commercially available potting soil in a climate-controlled chamber with a 16 h photoperiod, 1 mmol (m^2^)^-1^ s^-1^ of photosynthetically-active radiation, a temperature cycle of 22°C/18°C (day/night) and 65% relative humidity. Twelve to fifteen day old-plants (15–25 cm high, 4 expanded leaves) were used in all experiments.

Eggs of *Spodoptera littoralis* Boisd. (Lepidoptera: Noctuidae) were obtained from Aventis (Frankfurt, Germany) and were reared on an artificial wheat germ diet (Heliothis mix, Stonefly Industries, Bryan, TX, USA) for about 10–15 d at 22°C under an illumination of 750 μmol (m^2^)^-1^ s^-1^. For the herbivory treatments, one third instar larva was enclosed on a single leaf in a small cage made out of a paperboard tube (2 cm diameter). All herbivory treatments were started in the afternoon between 4 pm and 5 pm and caterpillars were allowed to feed for 19 hours. After removal of the caterpillars the plant material was immediately frozen in liquid nitrogen.

### Head-space volatile collection from intact plants

Head-space volatile collection was performed as described by Kunert et al. [43]. Plants were separately placed in 3 l glass desiccators which were tightly closed. Purified air pumped into the desiccator at a rate of 2 l min^-1^ came into contact with the plant and left the vessel through a filter packed with 30 mg SuperQ (ARS, Inc., Gainsville, USA). Volatiles were collected for 4 h (10 am – 2 pm). After the collection the plant material was immediately frozen in liquid nitrogen for further analysis. The volatile compounds were desorbed from the filters by eluting the filter twice with 100 μl dichloromethane containing nonyl acetate as an internal standard (10 ng μl^-1^).

### Volatile collection from macerated leaf samples

Frozen leaf material was pulverized in a mortar and an aliquot of 0.2 g plant powder placed in a glass vial with a septum in the lid. An 100 μm PDMS solid-phase microextraction (SPME) fiber (Supelco, Bellefonte, PA, USA) was inserted through the septum and exposed for 60 min at 40°C. The compounds adsorbed onto the fiber were analyzed by GC-MS. Approximate quantification was carried out with an external standard by performing analyses on 0.2 g of powdered tissue from control plants producing no volatile sesquiterpenes spiked with known amounts (4.5 ng, 9.0 ng, 45 ng and 90 ng) of (*E*)-β-caryophyllene (Sigma-Aldrich, Taufkirchen, Germany). Response factors for other compounds were determined by comparing signal intensity relative to (*E*)-β-caryophyllene using GC-FID with the detector operated at 250°C.

### Gas chromatography

A Hewlett-Packard model 6890 gas chromatograph was employed with the carrier gas He at 1 ml min^-1^, splitless injection (injector temperature, 220°C), a Chrompack CP-SIL-5 CB-MS column ((5%-phenyl)-methylpolysiloxane, 25 m x 0.25 mm i.d. x 0.25 μ film thickness, Varian, Palo Alto, CA, USA) and a temperature program from 40°C (3 min hold) at 5°C min^-1^ to 240°C (3 min hold). The coupled mass spectrometer was a Hewlett-Packard model 5973 with a quadrupole mass selective detector, transfer line temperature 230°C, source temperature 230°C, quadrupole temperature 150°C, ionization potential 70 eV and a scan range of 40–350 atomic mass units. Products were identified by comparison of retention times and mass spectra with those of authentic reference compounds.

### Measurement of plant hormones in maize leaf tissue

The analysis of plant hormones followed the procedure described in Wang et al. [44]. Tissue samples of 250 mg were harvested in FastPrep tubes containing 0.9 g of FastPrep matrix (BIO 101, Vista, CA, USA). Ethyl acetate (1 ml), spiked with 200 ng of D_2_-jasmonic acid, 40 ng D_4_-salicylic acid, and 40 ng jasmonic acid-^13^C_6_-isoleucine was added to each sample and then homogenized on a FastPrep homogenizer (Savant Instruments, Holbrook, NY, USA). After centrifugation at 12,000*g* for 20 min at 4°C, supernatants were transferred to fresh 1.5 ml Eppendorf tubes. The pellets were re-extracted with 1 ml of ethyl acetate and centrifuged. The combined supernatants were then evaporated to dryness under a vacuum. The residue was resuspended in 0.5 ml of 70% methanol/water (v/v), mixed for 5 minutes and centrifuged. The supernatants were analyzed by HPLC–MS/MS. Measurements were conducted on a 1200L LC/MS system (Varian). At a flow rate of 0.1 ml min^-1^, 10 μl of each sample was injected onto a ProntoSIL C18-ace-EPS column (50 x 2 mm, 5 μm, 120 Å, Bischoff, Leonberg, Germany). The mobile phase was composed of solvent A (0.05% formic acid) and solvent B (methanol) and was used in a gradient mode for the separation. The compounds were detected in the ESI negative mode and quantified as described in Wang et al. [44].

### RNA Hybridization

Plant RNA was prepared with the RNeasy plant mini kit (Qiagen, Hilden, Germany) according the manufacturer’s instructions. To verify an even loading of RNA, the gels were stained with ethidium bromide and visualized. A 400 bp fragment containing the first two exons of *tps23* was used as a probe, generated by linear PCR with the primer 5^′^- GAACTTCAAAAATACATCAGA-3^′^ and the complete ORF [15] as a template. The ORF of *tps10* was amplified from the pASK-IBA7 construct [14] with the primers forward: 5^′^-ATGGTAACCTGCATTAGCGCATGGATGCCACCGCCTTCCAC-3^′^ and reverse: 5^′^-ATGGTAACCTGCATTATATCAGAATAATGATATTGGATCCACAAAGA-3^′^. This fragment was used as a template for synthesis of a 1,196-bp probe by linear PCR with the primer 5^′^-TACTTGAAAGGCTCCCACC-3^′^. The probes were labeled with ^32^P-adenosine triphosphate using the Strip-EZ PCR procedure (Ambion, TX, USA). Blotting on a Nytran-Plus nylon membrane (Schleicher & Schuell, Germany), hybridization and washing were carried out following standard procedures. The blots were scanned with a Storm 840 Phosphoimager (Molecular Dynamics, Sunnyvale, CA, USA). Since a BLAST analysis of the recently sequenced maize genome revealed no *tps* genes with a high similarity to *tps10* and *tps23*, a potential cross-hybridization of the Northern probes is unlikely.

### Statistical Analysis

Statistical significance of associated samples was analyzed by a One Way Repeated Measures ANOVA or a Two Way ANOVA for independent samples. Normality of data was verified using the Kolmogorov-Smirnov test, while equality of variances was tested using a Leveen test. In case of non-normality and/or unequal variances, data were log_10_-transformed or analyzed by a Mann–Whitney rank sum test. Significant differences in the data are indicated for p<0.05.

## Competing interests

The authors declare that they have no competing interests.

## Authors’ contributions

TGK, CS, CL, SK and PL carried out the experimental work. CL performed the statistical analysis. JD and JG participated in the design of the study and improved the manuscript. TGK conceived of the study and drafted the manuscript. All authors read and approved the final manuscript.

## Supplementary Material

Additional file 1: Figure S1 Comparison of sesquiterpene emission from intact plants as measured by standard headspace volatile collection vs. SPME collection from macerated tissue. The results of the two methods are directly correlated with each other in terms of the amount emitted and the relative proportion of individual sesquiterpenes. (**A**) Maize plants were treated with *Spodoptera littoralis* caterpillars (see Methods). Headspace collection was carried out using a standard system as described in the Methods. For maceration-SPME, the plants were frozen right after volatile collection and ground in liquid nitrogen. Sesquiterpenes released from plant powder were collected using SPME. Samples were analyzed using GC-MS and the peak areas for (*E*)-β-farnesene + (*E*)-α-bergamotene (TPS10 products) and (*E*)-β-caryophyllene (TPS23 product) were used for comparison. To cover a broad range of volatile emission, plants of the inbred line B73 (which are known to emit only low amounts of sesquiterpenes) as well as plants of the hybrid Delprim (which are described as strong emitters) were used in this experiment. (**B**) GC-MS traces of sesquiterpenes emitted from an intact herbivore-induced Delprim plant (left) and sesquiterpenes released from powder of the same plant (right). 1, (*E*)-β-caryophyllene ; 2, (*E*)-α-bergamotene ; 3, (*E*)-β-farnesene ; 4, α-humulene; 5, β-bisabolene ; 6, β-sesquiphellandrene. Click here for file

## References

[B1] Raguso RA Wake Up and Smell the Roses: The Ecology and Evolution of Floral Scent Annu Rev Ecol Evol Syst 2008 39 549 569 10.1146/annurev.ecolsys.38.091206.095601

[B2] Unsicker SB Kunert G Gershenzon J Protective perfumes: the role of vegetative volatiles in plant defense against herbivores Curr Opin Plant Biol 2009 12 4 479 485 10.1016/j.pbi.2009.04.001 19467919

[B3] Huang M Sanchez-Moreiras AM Abel C Sohrabi R Lee S Gershenzon J Tholl D The major volatile organic compound emitted from Arabidopsis thaliana flowers, the sesquiterpene (E)-beta-caryophyllene, is a defense against a bacterial pathogen New Phytol 2012 193 4 997 1008 10.1111/j.1469-8137.2011.04001.x 22187939

[B4] Heil M Ton J Long-distance signalling in plant defence Trends Plant Sci 2008 13 6 264 272 10.1016/j.tplants.2008.03.005 18487073

[B5] Turlings TCJ Tumlinson JH Lewis WJ Exploitation of herbivore-induced plant odors by host-seeking parasitic wasps Science 1990 30 1251 1253 17829213 10.1126/science.250.4985.1251

[B6] Hoballah MEF Turlings TCJ Experimental evidence that plants under caterpillar attack may benefit from attracting parasitoids Evol Ecol Res 2001 3 5 553 565

[B7] Hoballah ME Köllner TG Degenhardt J Turlings TCJ Costs of induced volatile production in maize Oikos 2004 105 1 168 180 10.1111/j.0030-1299.2004.12831.x

[B8] Degen T Dillmann C Marion-Poll F Turlings TCJ High genetic variability of herbivore-induced volatile emission within a broad range of maize inbred lines Plant Physiol 2004 135 4 1928 1938 10.1104/pp.104.039891 15299140 PMC520764

[B9] Gouinguene S Degen T Turlings TCJ Variability in herbivore-induced odour emissions among maize cultivars and their wild ancestors (teosinte) Chemoecology 2001 11 9 16 10.1007/PL00001832

[B10] Köllner TG Schnee C Gershenzon J Degenhardt J The sesquiterpene hydrocarbons of maize (Zea mays) form five groups with distinct developmental and organ-specific distribution Phytochemistry 2004 65 13 1895 1902 10.1016/j.phytochem.2004.05.021 15279995

[B11] Köllner TG Schnee C Gershenzon J Degenhardt J The variability of sesquiterpenes emitted from two Zea mays cultivars is controlled by allelic variation of two terpene synthase genes encoding stereoselective multiple product enzymes Plant Cell 2004 16 5 1115 1131 10.1105/tpc.019877 15075399 PMC423204

[B12] Turlings TCJ Tumlinson JH Systemic release of chemical signals by herbivore-injured corn Proc Natl Acad Sci USA 1992 1 8399 8402 11607325 10.1073/pnas.89.17.8399 PMC49926

[B13] Schnee C Köllner TG Held M Turlings TCJ Gershenzon J Degenhardt J The products of a single maize sesquiterpene synthase form a volatile defense signal that attracts natural enemies of maize herbivores Proc Natl Acad Sci USA 2006 103 4 1129 1134 10.1073/pnas.0508027103 16418295 PMC1347987

[B14] Köllner TG Held M Lenk C Hiltpold I Turlings TCJ Gershenzon J Degenhardt J A maize (E)-beta-caryophyllene synthase implicated in indirect defense responses against herbivores is not expressed in most American maize varieties Plant Cell 2008 20 2 482 494 10.1105/tpc.107.051672 18296628 PMC2276456

[B15] Rasmann S Köllner TG Degenhardt J Hiltpold I Toepfer S Kuhlmann U Gershenzon J Turlings TCJ Recruitment of entomopathogenic nematodes by insect-damaged maize roots Nature 2005 434 7034 732 737 10.1038/nature03451 15815622

[B16] Howe GA Jander G Plant immunity to insect herbivores Annu Rev Plant Biol 2008 59 41 66 10.1146/annurev.arplant.59.032607.092825 18031220

[B17] Schmelz EA Alborn HT Banchio E Tumlinson JH Quantitative relationships between induced jasmonic acid levels and volatile emission in Zea mays during Spodoptera exigua herbivory Planta 2003 216 4 665 673 12569409 10.1007/s00425-002-0898-y

[B18] Schmelz EA Alborn HT Tumlinson JH Synergistic interactions between volicitin, jasmonic acid and ethylene mediate insect-induced volatile emission in Zea mays Physiol Plant 2003 117 3 403 412 10.1034/j.1399-3054.2003.00054.x 12654041

[B19] Schmelz EA Alborn HT Engelberth J Tumlinson JH Nitrogen deficiency increases volicitin-induced volatile emission, jasmonic acid accumulation, and ethylene sensitivity in maize Plant Physiol 2003 133 1 295 306 10.1104/pp.103.024174 12970495 PMC196606

[B20] Schmelz EA Alborn HT Tumlinson JH The influence of intact-plant and excised-leaf bioassay designs on volicitin- and jasmonic acid-induced sesquiterpene volatile release in Zea mays Planta 2001 214 2 171 179 10.1007/s004250100603 11800380

[B21] Reymond P Farmer EE Jasmonate and salicylate as global signals for defense gene expression Curr Opin Plant Biol 1998 1 5 404 411 10.1016/S1369-5266(98)80264-1 10066616

[B22] Staudt M Lhoutellier L Volatile organic compound emission from holm oak infested by gypsy moth larvae: evidence for distinct responses in damaged and undamaged leaves Tree Physiol 2007 27 10 1433 1440 10.1093/treephys/27.10.1433 17669734

[B23] Arimura GI Kopke S Kunert M Volpe V David A Brand P Dabrowska P Maffei ME Boland W Effects of feeding Spodoptera littoralis on lima bean leaves: IV Diurnal and nocturnal damage differentially initiate plant volatile emission Plant Physiol 2008 146 3 965 973 10.1104/pp.107.111088 18165324 PMC2259069

[B24] Hiltpold I Erb M Robert CAM Turlings TCJ Systemic root signalling in a belowground, volatile-mediated tritrophic interaction Plant Cell Environ 2011 34 8 1267 1275 10.1111/j.1365-3040.2011.02327.x 21477121

[B25] Lu S Xu R Jia JW Pang JH Matsuda SPT Chen XY Cloning and functional characterization of a beta-pinene synthase from Artemisia annua that shows a circadian pattern of expression Plant Physiol 2002 130 1 477 486 10.1104/pp.006544 12226526 PMC166579

[B26] Dudareva N Martin D Kish CM Kolosova N Gorenstein N Faldt J Miller B Bohlmann J (E)-beta-ocimene and myrcene synthase genes of floral scent biosynthesis in snapdragon: Function and expression of three terpene synthase genes of a new terpene synthase subfamily Plant Cell 2003 15 5 1227 1241 10.1105/tpc.011015 12724546 PMC153728

[B27] Aharoni A Giri AP Deuerlein S Griepink F de Kogel WJ Verstappen FWA Verhoeven HA Jongsma MA Schwab W Bouwmeester HJ Terpenoid metabolism in wild-type and transgenic Arabidopsis plants Plant Cell 2003 15 12 2866 2884 10.1105/tpc.016253 14630967 PMC282818

[B28] Gouinguene SP Turlings TCJ The effects of abiotic factors on induced volatile emissions in corn plants Plant Physiol 2002 129 3 1296 1307 10.1104/pp.001941 12114583 PMC166523

[B29] Pare PW Tumlinson JH De novo biosynthesis of volatiles induced by insect herbivory in cotton plants Plant Physiol 1997 114 4 1161 1167 12223763 10.1104/pp.114.4.1161 PMC158408

[B30] Ryan CA The systemin signaling pathway: differential activation of plant defensive genes Biochimica Et Biophysica Acta-Protein Structure and Molecular Enzymology 2000 1477 1–2 112 121 10.1016/s0167-4838(99)00269-1 10708853

[B31] Ryan CA Pearce G Scheer J Moura DS Polypeptide hormones Plant Cell 2002 14 S 251 264 10.1105/tpc.010484 PMC151259 12045281

[B32] Robert HS Friml J Auxin and other signals on the move in plants Nat Chem Biol 2009 5 5 325 332 10.1038/nchembio.170 19377459

[B33] Farmer EE Almeras E Krishnamurthy V Jasmonates and related oxylipins in plant responses to pathogenesis and herbivory Curr Opin Plant Biol 2003 6 4 372 378 10.1016/S1369-5266(03)00045-1 12873533

[B34] Wasternack C Jasmonates: An update on biosynthesis, signal transduction and action in plant stress response, growth and development Ann Bot 2007 100 681 697 10.1093/aob/mcm079 17513307 PMC2749622

[B35] Boland W Hopke J Donath J Nuske J Bublitz F Jasmonic acid and coronatin induce odor production in plants Angewandte Chemie-International Edition in English 1995 34 15 1600 1602 10.1002/anie.199516001

[B36] Zhang ZP Baldwin IT Transport of 2-C-14 jasmonic acid from leaves to roots mimics wound-induced changes in endogenous jasmonic acid pools in Nicotiana sylvestris Planta 1997 203 4 436 441 10.1007/s004250050211

[B37] Thorpe MR Ferrieri AP Herth MM Ferrieri RA C-11-imaging: methyl jasmonate moves in both phloem and xylem, promotes transport of jasmonate, and of photoassimilate even after proton transport is decoupled Planta 2007 226 2 541 551 10.1007/s00425-007-0503-5 17356850

[B38] Wasternack C Stenzel I Hause B Hause G Kutter C Maucher H Neumerkel J Feussner I Miersch O The wound response in tomato - Role of jasmonic acid J Plant Physiol 2006 163 3 297 306 10.1016/j.jplph.2005.10.014 16368162

[B39] Engelberth J Seidl-Adams I Schultz JC Tumlinson JH Insect elicitors and exposure to green leafy volatiles differentially upregulate major octadecanoids and transcripts of 12-oxo phytodienoic acid reductases in Zea mays Mol Plant Microbe Interact 2007 20 6 707 716 10.1094/MPMI-20-6-0707 17555278

[B40] Engelberth J Contreras CF Viswanathan S Transcriptional analysis of distant signaling induced by insect elicitors and mechanical wounding in Zea mays PLoS One 2012 7 4 e34855 e34855 10.1371/journal.pone.0034855 22511969 PMC3325234

[B41] Koo AJK Gao X Jones AD Howe GA A rapid wound signal activates the systemic synthesis of bioactive jasmonates in Arabidopsis Plant J 2009 59 6 974 986 10.1111/j.1365-313X.2009.03924.x 19473329

[B42] Zimmermann MR Maischak H Mithoefer A Boland W Felle HH System potentials, a novel electrical long-distance apoplastic signal in plants, induced by wounding Plant Physiol 2009 149 3 1593 1600 10.1104/pp.108.133884 19129416 PMC2649404

[B43] Kunert G Reinhold C Gershenzon J Constitutive emission of the aphid alarm pheromone, (E)-beta-farnesene, from plants does not serve as a direct defense against aphids BMC Ecol 2010 10 23-Article No 23 21092302 10.1186/1472-6785-10-23 PMC3002888

[B44] Wang L Halitschke R Kang JH Berg A Harnisch F Baldwin IT Independently silencing two JAR family members impairs levels of trypsin proteinase inhibitors but not nicotine Planta 2007 226 1 159 167 10.1007/s00425-007-0477-3 17273867

