# Supplementary material for: Localization of sesquiterpene formation and emission in maize leaves after herbivore damage
Source: BMC Plant Biol. 2013 Jan 30;13:15. doi: 10.1186/1471-2229-13-15 (PMC3570303; doi:10.1186/1471-2229-13-15)
Supplement: Additional file 1: Figure S1 — Comparison of sesquiterpene emission from intact plants as measured by standard headspace volatile collection vs. SPME collection from macerated tissue. The results of the two methods are directly correlated with each other in terms of the amount emitted and the relative proportion of individual sesquiterpenes. (A) Maize plants were treated with Spodoptera littoralis caterpillars (see Methods). Headspace collection was carried out using a standard system as described in the Methods. For maceration-SPME, the plants were frozen right after volatile collection and ground in liquid nitrogen. Sesquiterpenes released from plant powder were collected using SPME. Samples were analyzed using GC-MS and the peak areas for (E)-β-farnesene + (E)-α-bergamotene (TPS10 products) and (E)-β-caryophyllene (TPS23 product) were used for comparison. To cover a broad range of volatile emission, plants of the inbred line B73 (which are known to emit only low amounts of sesquiterpenes) as well as plants of the hybrid Delprim (which are described as strong emitters) were used in this experiment. (B) GC-MS traces of sesquiterpenes emitted from an intact herbivore-induced Delprim plant (left) and sesquiterpenes released from powder of the same plant (right). 1, (E)-β-caryophyllene ; 2, (E)-α-bergamotene ; 3, (E)-β-farnesene ; 4, α-humulene; 5, β-bisabolene ; 6, β-sesquiphellandrene. [file 1471-2229-13-15-S1.ppt]

## Slide 1
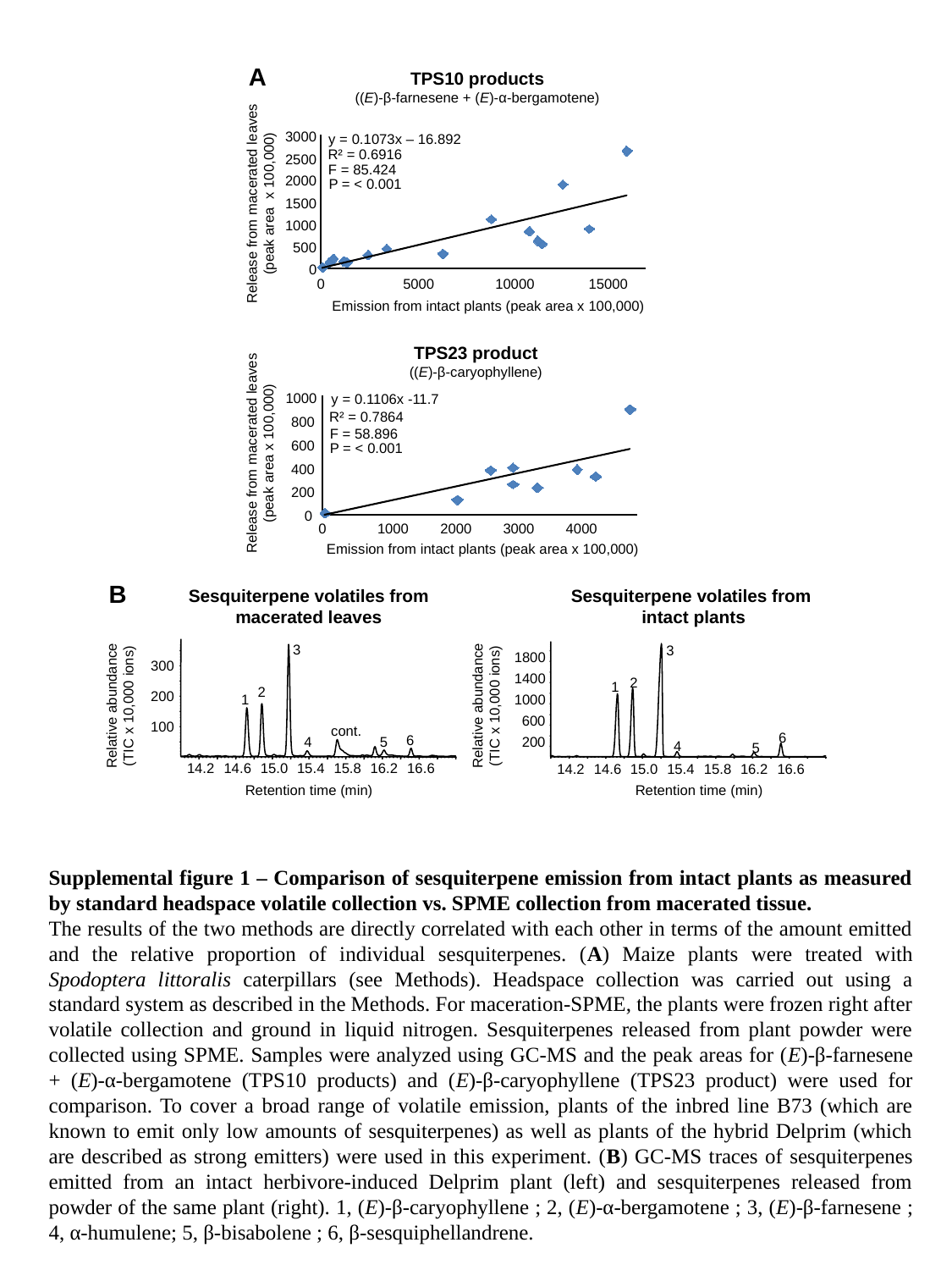

A
TPS10 products
((E)-β-farnesene + (E)-α-bergamotene)
3000
y = 0.1073x – 16.892
R² = 0.6916
2500
F = 85.424
2000
P = < 0.001
Release from macerated leaves
(peak area x 100,000)
1500
1000
500
0
0
5000
10000
15000
Emission from intact plants (peak area x 100,000)
TPS23 product
((E)-β-caryophyllene)
1000
y = 0.1106x -11.7
R² = 0.7864
800
F = 58.896
Release from macerated leaves
(peak area x 100,000)
600
P = < 0.001
400
200
0
0
1000
2000
3000
4000
Emission from intact plants (peak area x 100,000)
B
Sesquiterpene volatiles from
macerated leaves
Sesquiterpene volatiles from
 intact plants
3
3
 1800
2
 1400
1
 1000
600
6
4
5
200
14.2
14.6
15.0
15.4
15.8
16.2
16.6
300
2
Relative abundance
(TIC x 10,000 ions)
Relative abundance
(TIC x 10,000 ions)
1
200
cont.
100
6
5
4
14.2
14.6
15.0
15.4
15.8
16.2
16.6
Retention time (min)
Retention time (min)
Supplemental figure 1 – Comparison of sesquiterpene emission from intact plants as measured by standard headspace volatile collection vs. SPME collection from macerated tissue.
The results of the two methods are directly correlated with each other in terms of the amount emitted and the relative proportion of individual sesquiterpenes. (A) Maize plants were treated with Spodoptera littoralis caterpillars (see Methods). Headspace collection was carried out using a standard system as described in the Methods. For maceration-SPME, the plants were frozen right after volatile collection and ground in liquid nitrogen. Sesquiterpenes released from plant powder were collected using SPME. Samples were analyzed using GC-MS and the peak areas for (E)-β-farnesene + (E)-α-bergamotene (TPS10 products) and (E)-β-caryophyllene (TPS23 product) were used for comparison. To cover a broad range of volatile emission, plants of the inbred line B73 (which are known to emit only low amounts of sesquiterpenes) as well as plants of the hybrid Delprim (which are described as strong emitters) were used in this experiment. (B) GC-MS traces of sesquiterpenes emitted from an intact herbivore-induced Delprim plant (left) and sesquiterpenes released from powder of the same plant (right). 1, (E)-β-caryophyllene ; 2, (E)-α-bergamotene ; 3, (E)-β-farnesene ; 4, α-humulene; 5, β-bisabolene ; 6, β-sesquiphellandrene.
